# Sarcoidosis Associated with Oxaliplatin-Based Chemotherapy for Colorectal Cancer

**DOI:** 10.1155/2014/203027

**Published:** 2014-03-04

**Authors:** Ji Hoon Choi, Jung A. Shin, Hye Kyeong Park, Su Young Kim, Hoon Jung, Sung-Soon Lee, Hye Ran Lee, Hyeon-Kyoung Koo

**Affiliations:** ^1^Department of Internal Medicine, Ilsan Paik Hospital, Inje University College of Medicine, Daehwa-dong, Ilsanseo-gu, Goyang-si, Gyeonggi-do 2240, Republic of Korea; ^2^Department of Radiology, Ilsan Paik Hospital, Inje University College of Medicine, Daehwa-dong, Ilsanseo-gu, Goyang-si, Gyeonggi-do 2240, Republic of Korea

## Abstract

Acute lung injury occasionally occurs after chemotherapy, but pulmonary toxicities by oxaliplatin-based chemotherapy have rarely been identified. A 76-year-old female with rectosigmoid colon cancer presented with ongoing dyspnea after the eighth cycle of standard chemotherapy (5-fluorouracil, sodium folinic acid, and oxaliplatin: FOLFOX). Nodular consolidation progressed despite antibiotics and BAL fluid analysis was compatible with the diagnosis of sarcoidosis. Corticosteroid therapy rapidly improved the symptoms and radiographic findings. We report this first case of secondary sarcoidosis related to FOLFOX therapy with review of references.

## 1. Introduction

The standard palliative treatment for patients with advanced or metastatic colorectal cancer, consisting of oxaliplatin or irinotecan and 5-fluorouracil (5-FU) based chemotherapy, can extend patient's survival up to 22 months [1–3]. The usual adverse effects following these regimens are hematological (13–52%), gastrointestinal (10–33%), and neurological (0–8%) toxicities [4]. However despite the widespread application of this regimen, pulmonary toxicities of oxaliplatin-based chemotherapy are rarely reported. We recently observed a rare case of secondary sarcoidosis related to oxaliplatin-based chemotherapy that presented as progressive dyspnea.

## 2. Case Report

**Figure 1:**
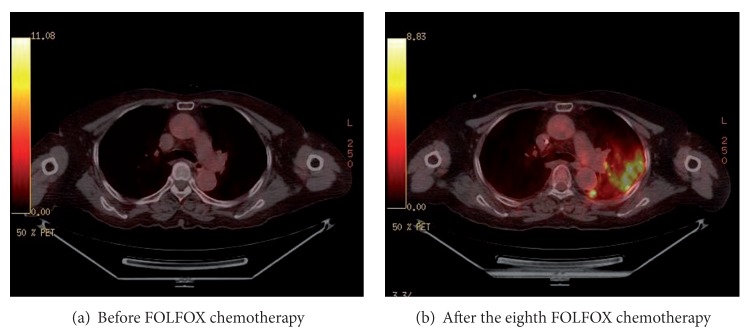
PET-CT after the eighth cycle of chemotherapy.

**Figure 2:**
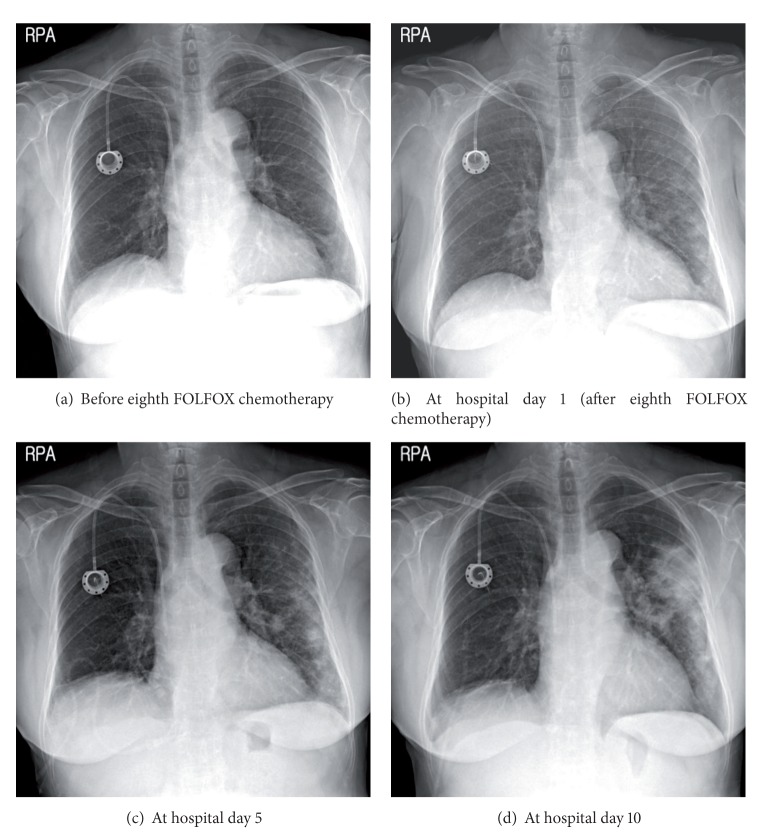
Serial changes in the chest X-ray during admission.

**Figure 3:**
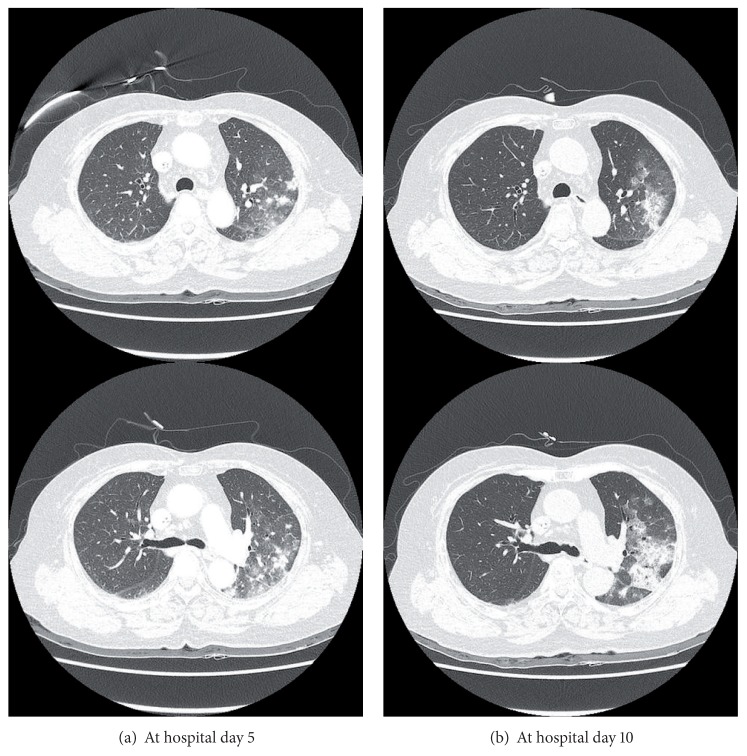
Chest CT findings of lung infiltration.

**Figure 4:**
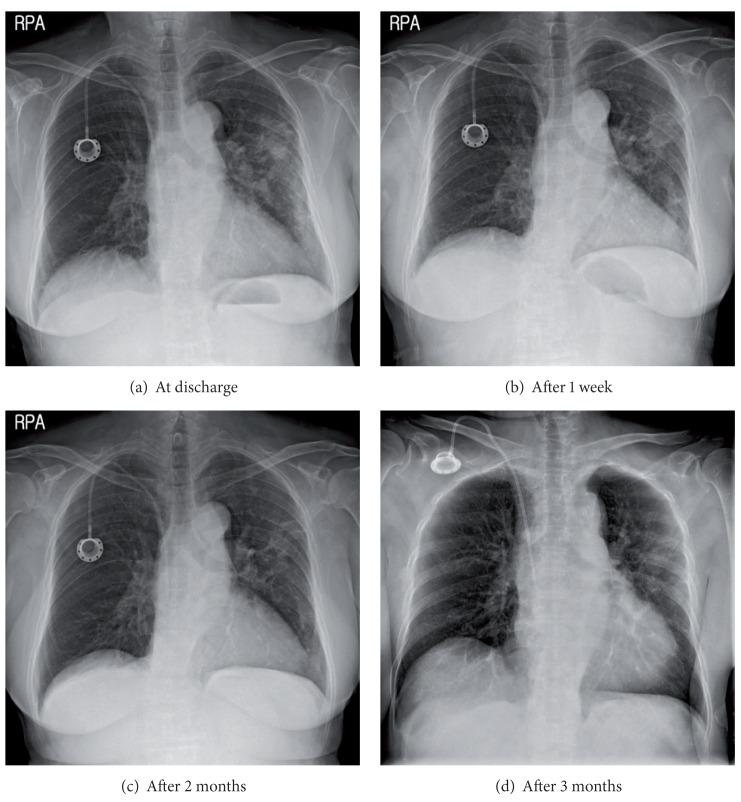
Resolution of chest X-ray after treatment for sarcoidosis.

A 76-year-old female never-smoker visited our hospital because of progressive dyspnea beginning one month ago. She had been diagnosed with rectosigmoid colon adenocarcinoma and had undergone low anterior resection and a total abdominal hysterectomy with bilateral salpingo-oophorectomy 8 months previously. Her surgical stage was T4bN0 because of direct invasion into the left ovary, but the initial computed tomography (CT) showed small uncertain nodules in liver and lung. After a second cycle of adjuvant 5-fluorouracil with leucovorin, positron-emission tomography (PET-CT) scan showed progression of liver metastasis with FDG-uptake (SUV 8.4), but the lung nodules in the left upper lobe were stable and without FDG-uptake. The chemotherapy regimen was changed to 5-fluorouracil, leucovorin, and oxaliplatin (FOLFOX) and infused every other week without significant adverse events. After the fourth cycle of FOLFOX regimen, asymptomatic pulmonary embolism was detected and anticoagulation with warfarin had started. At completion of the eighth cycle, she complained of progressive dyspnea without other respiratory symptoms. In PET-CT, the size of previous pulmonary nodules had been decreased, but several other ill-defined nodules that showed FDG-uptake (SUV 9.0–11.8) had developed in the left upper lobe and left lower lobe (Figure 1). She was admitted to hospital because of hypoxia, and her oxygen needs increased as time passed. Crackles were present at the left side lower lung field. Her blood pressure was 140/80 mmHg; temperature was 36.5°C; pulse rate was 90 beats/min and the respiration rate was 22/min. A simple chest radiograph showed newly appeared nodules which progressed to consolidation with ground glass opacities day by day despite empirical antibiotic therapy (Figures 2 and 3). The extent of consolidation spread to adjacent areas but remained localized to the left side of the lung. She was not febrile and laboratory markers for inflammation were not elevated (leukocyte 7730/*μ*L, CRP 0.7 mg/dL). At the 10th day in hospital, she underwent bronchoalveolar lavage, but a transbronchial lung biopsy was not performed because she was under anticoagulation. Since we suspected drug-induced lung reaction to chemotherapy, steroid treatment (1 mg/kg/day) was begun just after bronchoscopy. The study revealed no infectious cause, with a total count of 50 cells/*μ*L (5% neutrophils, 86% lymphocytes, without eosinophil or basophil), and CD4/CD8 ratio was 3.4. Negative results were obtained for the polymerase chain reaction for *pneumocystis jiroveci, Mycobacterium tuberculosis* and respiratory viruses, and for cultures for common bacteria, acid-fast bacilli, and fungi. No malignant cells were observed at cytologic examination and the BAL fluid carcinoembryonic antigen level was 23.6. Following the report for the CD4/CD8 count, serum angiotensin converting enzyme (ACE) level was checked, and was found elevated to 52.5 U/L even though steroid had been preadministered for five days. Autoantibodies such as antinuclear antibody, rheumatoid factor, and antineutrophil cytoplasmic antibody were all negative. After steroid treatment, the dyspnea and infiltration apparent on chest X-rays began to improve from the following day, and she was discharged from the hospital one week later (Figure 4). After two months, the radiographic infiltration had nearly disappeared (Figure 4), and level of ACE falls to 10.4 U/L.

## 3. Discussion

Acute lung injury after chemotherapy occasionally occurs, but pulmonary toxicities due to oxaliplatin-based chemotherapy had been infrequently reported in a small number of case reports [5–13]. Pulmonary complications by this regimen had been described as heterogeneous clinical course, histopathologic features, and prognosis. The presumed diagnosis of lung toxicities included organizing pneumonia [5–9], diffuse alveolar damage [6, 10–12], nonspecific interstitial pneumonia [7], eosinophilic pneumonia [13], and usual interstitial pneumonia. The interval from the initial chemotherapy to the lung injury varied from one day [6] to more than 6 months [7], and the overall mortality was around 30%. Our case demonstrated sarcoidosis secondary to oxaliplatin-based chemotherapy which developed 3 months after the exposure to oxaliplatin.

Sarcoidosis is a multisystemic inflammatory disease characterized by the formation of noncaseating granulomas that commonly affect the lungs and the lymphatic system but can involve any other organs [14]. Most of pulmonary sarcoidosis is accompanied by systemic or mediastinal lymph node enlargement. However about 10% of pulmonary sarcoidosis presents as nonspecific lung infiltration without lymph node enlargement and cannot be distinguished from other idiopathic interstitial lung diseases by radiographic findings.

Many studies have tried to elucidate the pathogenesis and etiology of sarcoidosis, but these remain unclear. Granulomatous lung disease can be caused by various agents such as interferon-*γ* therapy for chronic hepatitis or multiple sclerosis [15, 16]; methotrexate or TNF-*α* blocking agent for autoimmune disease [17–19]; BCG [20]; and some antineoplastic drugs including everolimus or gefitinib [21]. However a sarcoidosis reaction complicated by FOLFOX chemotherapy has never been reported previously. The case described in this study is the first report of sarcoidosis secondary to oxaliplatin-based chemotherapy.

Diagnosis of sarcoidosis can be established when compatible clinical features are present together with supporting laboratory, radiologic, and pathologic findings. In addition alterative disease forming granuloma must be excluded. Revealing of noncaseating granuloma by biopsy is important for diagnosis of sarcoidosis, but several supportive tests can enhance the diagnostic probability. These include elevated serum ACE level [22] and BAL lymphocytosis with elevated CD4/CD8 ratio greater than 3.5–4.0 [23–25]. Even though biopsy was not performed due to anticoagulation in our patient, we were able to diagnose sarcoidosis by typical BAL fluid findings and with compatible clinical, laboratory, and radiological findings. Elevated serum ACE level usually represents sarcoidosis with high negative predictive values, but the ACE level can also rise in several circumstances, such as Gaucher's disease, tuberculosis, leprosy, histoplasmosis, untreated hyperthyroidism, psoriasis, and lymphoma [26]. In our case, the patient had no other features suggestive of these conditions, and there was no evidence of systemic granulomatous disease in PET-CT and infectious organisms in BAL fluid analysis. Furthermore, the serum ACE level declined after treatment for sarcoidosis. Serum ACE level is well known to reflect disease activities and used to monitor the treatment effects in clinical practice.

Our patient had complained of progressive dyspnea after completion of the eighth FOLFOX regimen. The sarcoidosis reaction had progressed despite discontinuation of chemotherapy, and the patient required steroid treatment. Our patient's course had shown several unusual features of secondary sarcoidosis due to oxaliplatin-based therapy. First, secondary sarcoidosis can occur at any time after initial application of FOLFOX, not just after the first administration. Second, this sarcoidosis could progress after cessation of the causative agent, and avoidance would not be sufficient for treatment.

In conclusion, although the exact mechanism of this injury should be evaluated further, FOLFOX chemotherapy can be a causative agent of secondary sarcoidosis and that withdrawal may not be sufficient for control of adverse events. Apart from common adverse events, lung toxicities, especially sarcoidosis, should also be considered in otherwise unexplained lung disease whenever patients are treated with oxaliplatin-based regimens.
